# Sex differences in thigh muscle volumes, sprint performance and mechanical properties in national-level sprinters

**DOI:** 10.1371/journal.pone.0224862

**Published:** 2019-11-05

**Authors:** Sergi Nuell, Víctor Illera-Domínguez, Gerard Carmona, Xavier Alomar, Josep Maria Padullés, Mario Lloret, Joan Aureli Cadefau

**Affiliations:** 1 Institut Nacional d’Educació Física de Catalunya (INEFC), Universitat de Barcelona, Barcelona (UB), Spain; 2 Futbol Club Barcelona (FCB), Barcelona, Spain; 3 Creu Blanca, Barcelona, Spain; Victoria University, AUSTRALIA

## Abstract

The purpose of this study was to determine and compare thigh muscle volumes (MVs), and sprint mechanical properties and performance between male and female national-level sprinters. We also studied possible relationships between thigh MVs and sprint performance. Nine male and eight female national-level sprinters participated in the study. T1-weighted magnetic resonance images of the thighs were obtained to determine MVs of quadriceps, hamstrings and adductors. Sprint performance was measured as the time to cover 40 and 80 m. Instantaneous sprint velocity was measured by radar to obtain theoretical maximum force (F0), theoretical maximum velocity (V0) and maximum power (Pmax). When MVs were normalized by height–mass, males showed larger hamstrings (13.5%, ES = 1.26, P < 0.05) compared with females, while quadriceps and adductors showed no statistically significant differences. Males were extremely faster than females in 40 m (14%, ES = 6.68, P < 0.001) and in 80 m (15%, ES = 5.01, P < 0.001. Males also showed increased sprint mechanical properties, with larger F_0_ (19%, ES = 1.98, P < 0.01), much larger P_max_ (46%, ES = 3.76, P < 0.001), and extremely larger V_0_ (23%, ES = 6.97, P < 0.001). With the pooled data, hamstring and adductor MVs correlated strongly (r = -0.685, P < 0.01) and moderately (r = -0.530, P < 0.05), respectively, with sprint performance; while quadriceps showed no association. The sex-stratified analysis showed weaker associations compared with pooled data, most likely due to small sample size. In conclusion, males were faster than females and showed larger MVs, especially in hamstrings. Moreover, regarding the thigh muscles, hamstrings MV seems the most related with sprint performance as previously proposed.

## Introduction

Sprint ability is one of the most highly appreciated individual qualities in a majority of sports. Many factors seem to influence sprint performance; however, it is believed that the ability to produce large propulsive forces is one of its strongest predictors [1–3]. The overall mechanical capacity to apply anteroposterior ground reaction forces when sprinting is portrayed by the linear force-velocity (F-V) and parabolic power-velocity (P-V) relationships, which characterize the mechanical limits of the entire neuromuscular system [2,4]. The P-F-V profile can be accurately computed from anthropometric (body mass and stature) and spatiotemporal variables (split times or instantaneous velocity), using a validated computerized method [2].

To move the body forward when running, humans have to produce a combination of lower-limb joint torques. Muscle volume (MV) is the major determinant of joint torque [5]; hence, identifying the key lower-limb muscles in sprinting is important for coaches and athletes. To date, many studies have investigated the relationship between lower-limb muscularity and sprint performance, leading to some controversy concerning the results [6–10]. For example, Sugisaki et al. [6] and Tottori et al. [7] concluded that having greater quadriceps and adductor muscles is advantageous for sprint performance. In contrast to those results, in recent work, Sugisaki et al. [9] found relationships between sprint performance and both hamstrings and glutes, but no association with quadriceps or adductors. Along the same lines, after comparing sprinters to non-sprinters, Ema et al. [10] reported strong correlations between hamstrings muscle volume and sprint performance. However, the vast majority of studies analyzing MVs in sprinters have been performed on males, and it seems there is a lack of muscularity studies in female sprinters.

A substantial gender difference in sprint performance is a common observation, regardless of the level: males are faster than females [11–13]. It is believed that most performance differences are due to variations in morphological and physiological characteristics typical of men and women [11]. Success in sprint is highly dependent on the generation of large forces over short periods of time [12,14–16]; thus, power production is believed to be a strong determinant of sprint performance [2–4,14]. In this regard, women tend to have a lower absolute muscle mass and smaller skeletons [12,17]. During sprinting, these anthropometric disparities could lead to differences in the capacity to produce large forward acceleration in order to achieve maximal speed [17]. To our knowledge very few studies focused on sex differences in muscularity in high-level sprinters. While it is known that male sprinters have more muscle mass than females [18], it remains to be established if this finding persists once muscle mass is normalized to heights and body mass. Hence, differences in normalized MVs between male and female high-level sprinters, and the relation between MV and performance are potentially a highly fertile field of investigation in order to elucidate sex differences in sprint performance.

Therefore, the purpose of this study was to determine and compare thigh MVs, and sprint mechanical properties and performance between male and female national-level sprinters. Additionally, the relationships between thigh MV and sprint performance were investigated.

## Methods

### Participants

Nine male (age 23.3 ± 1.7 years; body mass 73.8 ± 8.6 kg; height 180.0 ± 7.3 cm) and eight female (age 23.9 ± 5.3 years; body mass 57.0 ± 6.9 kg; height 163.3 ± 7.9 cm) national-level sprinters participated in this study. They were specialists at sprinting from 100 m to 400 m, and they had performed competitive sprinting for at least 4 years. (Table 1) Eight skinfold thicknesses were measured to estimate the percentage of body fat using the equations published by Withers et al. [19]. All the measurements in the present study were obtained during the off-season. All the participants gave informed written consent to participate in the study, which was conducted in accordance with the Declaration of Helsinki and was approved by the Ethics Committee of the Catalan Sports Council.

**Table 1:** Sprinter’s individual sex and event characteristics.

Participant	Sex	Event	Event PB (s)
01	Female	400 m	58.39
02	Female	400 m	58.97
03	Female	200 m	24.98
04	Female	200 m	25.32
05	Female	200 m	26.87
06	Female	400 m	59.19
07	Female	100 m	12.07
08	Female	100 m	11.94
09	Male	100 m	11.21
10	Male	100 m	10.95
11	Male	200 m	21.13
12	Male	400 m	48.14
13	Male	100 m	11.06
14	Male	100 m	10.71
15	Male	200 m	20.97
16	Male	400 m	47.28
17	Male	100 m	10.45

### Design

A cross-sectional study was designed to investigate differences in sprint performance, thigh MVs and sprint mechanical properties between male and female national-level sprinters, and examine possible relationships between these factors. All the testing occurred at the very beginning of the pre-season, when hypertrophic or performance adaptations seem to not have occurred yet [20]. Magnetic resonance imaging analysis were performed after at least 72 h of rest, to avoid any influence of acute muscle swelling [21]. Meanwhile, sprint tests were conducted after at least 48 h of rest. All the sprint tests were performed over a tartan surface of a track in a stadium. The 40 m sprint and sprint mechanical properties tests were recorded during the same sprint. In all the sprint tests, the athletes adopted the three-point start position, using sprint spikes. Moreover, in order to achieve a faster and more reliable start, they were told to start at will, when they wanted. The sprint tests were performed on two different days.

### Muscle volumes

A series of cross-sectional images of each subject’s thighs were obtained by magnetic resonance imaging with an Avanto 1.5T (Magnetom Avanto, Siemens Healthineers, Germany). Transaxial images (slice thickness, 2 mm; increment, 2 mm) were acquired in a 320x320 matrix, 40x40 field of view in 20-second blocks. The volunteers were placed supine inside the scanner with their heads outside the MR bore, with their thighs covered with a leg coil, while their limb position was fixed through the use of a custom-made foot restraint. Between 280 and 320 images (depending on each subject’s thigh length) were obtained for each leg from the iliac spine to the patella.

**Fig 1:**
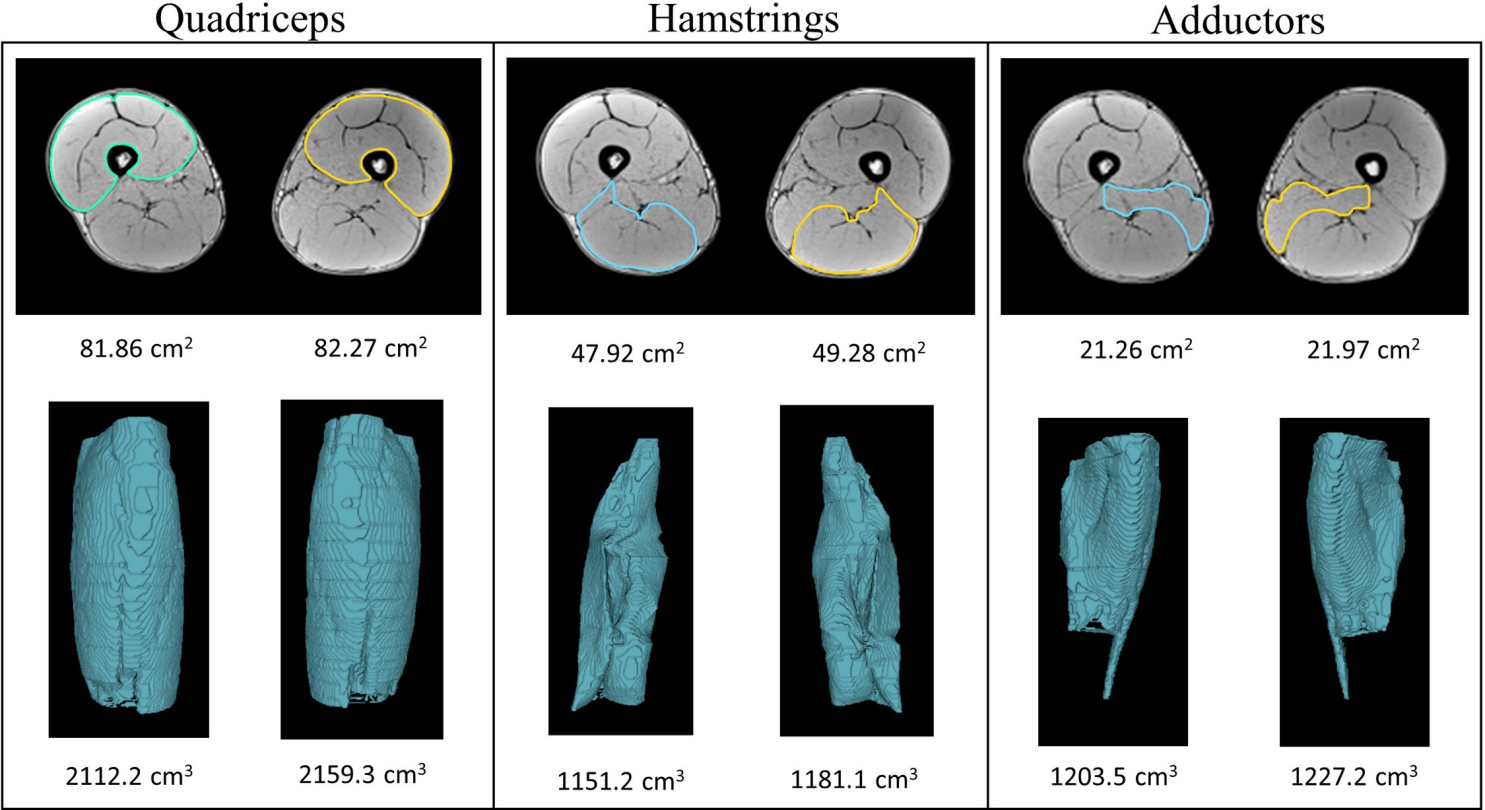
Quadriceps, hamstring and adductor muscle volume assessment. Representative magnetic resonance imaging scans, mid-thigh, of subject no. 10 (male) used for measuring cross-sectional area of quadriceps, hamstrings and adductors, and their respective muscle volume reconstructions.

Vastus lateralis, vastus intermedius, vastus medialis and rectus femoris were outlined together and identified as quadriceps. Biceps femoris long head, biceps femoris short head, semitendinosus and semimembranosus were outlined together and identified as hamstrings. The same approach was adopted for the assessment of adductor MV, which includes pectineus, adductor longus, brevis, magnus and gracilis. The edges of the quadriceps, hamstrings and adductors were manually outlined, image by image and by the same researcher, following the reference method proposed by Nordez et al. [22] using Osirix 8.5.2 (Pixmeo, Geneva, Switzerland). The total volume of each muscle was calculated from the range between the last image where the ischial tuberosity was visible and the last image where the muscle was visible (Fig 1). MV results are shown as the average of right and left thighs. The intra-investigator coefficient of variation for muscle segmentation was 1.1% ± 0.4% for the muscles assessed; similar to previous estimations of error for this method [22,23].

To reduce the effects of body size on muscle size differences between males and females, MVs were normalized by body size metrics. Lower-limb MVs have been shown to vary with the product of height and body mass in healthy active subjects [18,24]. Thus, MVs were normalized by height–mass.

### Sprint performance

Sprint tests were carried out on two different days, separated by 48 h. On day one, sprinters performed two maximal 40 m sprints with 6 min of rest between them; and on day two, they performed two maximal 80 m sprints with 15 min of rest. After 40 min of standardized warm-up and activation conducted by their coach, the athletes started the tests. Two pairs of Microgate Witty photocells (Microgate, Italy) were placed at distances of 0 and 40 m, and at 0 and 80 m, for the 40 m and 80 m sprint, respectively. The best time over each distance was used for further analysis. The sprinters started their attempts just before the first photocell, to start the timer with their very first movement.

### Sprint mechanical properties

Instantaneous velocity over the 40 m sprint was measured by radar (Stalker ATSII, Plano, Texas), as previously validated in human sprint experiments [2]. The device was placed on a tripod 1.5 m behind the subject at a height of 1 m: approximately the average subject’s center of gravity. Subjects were instructed to hold their three-point starting position static for about one second, and then to start running forward without any countermovement. As mentioned, the fastest trial, based on the photocell times, was selected for further analysis. The resultant data were subsequently analyzed using the simple field method validated by Samozino et al. [2]. Briefly, this computational method is based on macroscopic inverse dynamics analysis of the center of gravity motion. Velocity–time data are fitted to an exponential function, after which instantaneous velocity is derived to compute the net horizontal anteroposterior ground reaction forces and power. Individual force–velocity relationships are then extrapolated to calculate F_0_ and V_0_ capabilities, and the underlying P_max_, as described elsewhere [2–4,17].

### Statistical analysis

Descriptive data are presented as mean ± SD. The normal distribution of the data was checked using the Shapiro-Wilk tests. An independent sample *t*-test was performed to compare the different variables between male and female sprinters accompanied with the absolute mean differences between groups presented with 95% confidence intervals (CI) (lower limit: upper limit). Effect sizes (ES) and 95% CI (lower limit: upper limit) were calculated to assess the magnitude of the difference between the means of the two groups using an ES calculator [25] as proposed by Ellis [26]. The thresholds for these ES values were: 0.2, trivial; 0.6, small; 1.2, moderate; 2.0, large; 4.0, very large; and > 4.0, extremely large, as recommended by Hopkins et al. [27] The Pearson correlation coefficient was employed to evaluate the association between the variables of interest of the pooled data, with thresholds of: 0.40–0.59, moderate; 0.60–0.79, strong; 0.80–1, very strong. The significance level was set at *P* < 0.05. Statistical analysis was performed using SPSS v.23.0.0.0 (IBM, Armonk, New York).

## Results

### Physical characteristics

Male and female sprinters were similar in age and body mass index. However, they presented significant differences in anthropometric characteristics such as height, mass and percentage of fat (Table 2).

**Table 2:** Physical characteristics.

	Females (n = 8)	Males (n = 9)
**Age (year)**	23.9 ± 5.3	23.3 ± 1.7
**Height (m)**	1.63 ± 0.08	1.80 ± 0.07**
**Mass (kg)**	57.0 ± 6.9	73.8 ± 8.6**
**Body mass index**	21.4 ± 1.4	22.6 ± 1.3
**Fat percentage (%)**	14.9 ± 2.9	9.2 ± 1.0**

Data are means ± SD.

** Significant differences between groups at *P* < 0.01.

### Muscle volumes

**Fig 2:**
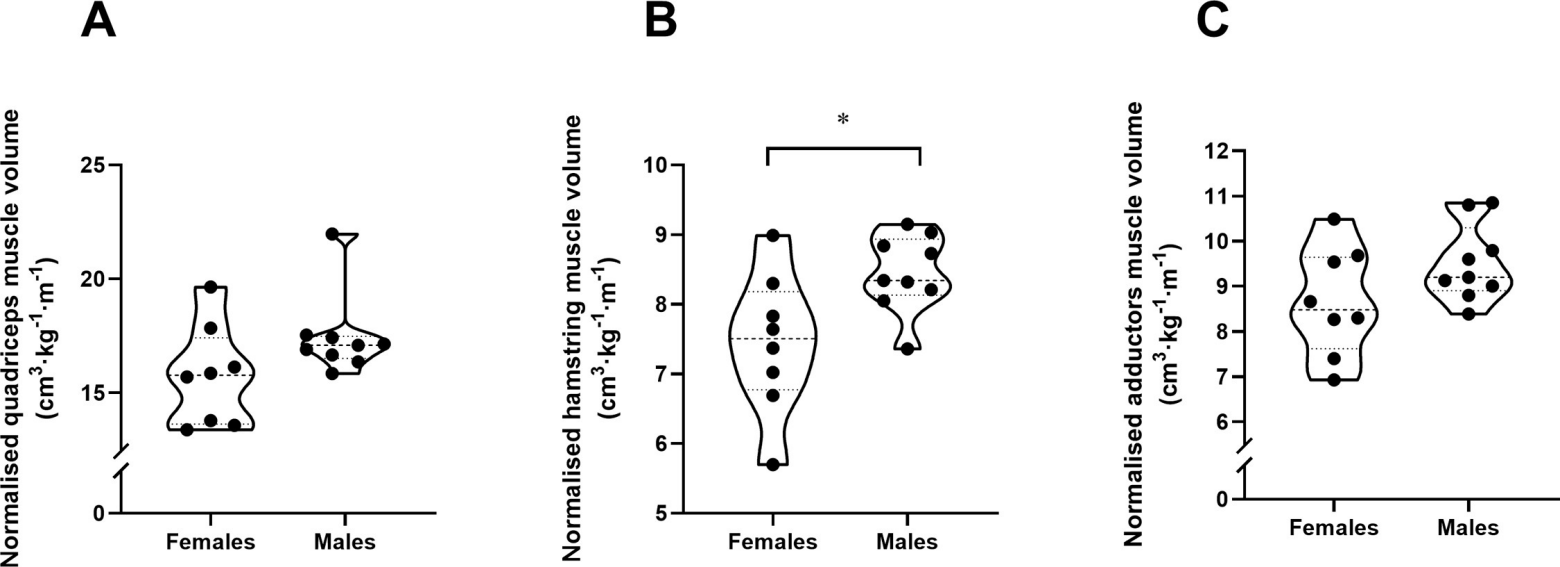
Muscle volumes. Normalized quadriceps (A), hamstrings (B) and adductors (C) muscle volumes between male and female sprinters. *Significant differences between groups at *P* < 0.05.

Due to differences in body size (Table 2) absolute MVs of the thigh were significantly higher in males than in females (58%-64%, *P* < 0.0001) (Table 3). However, when normalized by height–mass the differences were not that evident. Males showed larger hamstrings (13.5%, *P* = 0.021), quadriceps (10.8%, *P* = 0.09) and adductors (9.8%, *P* = 0.11), although the differences in quadriceps and adductors did not reach statistical significance (Table 3 and Fig 2).

**Table 3:** Values of variables analyzed.

	Females (n = 8)	Males (n = 9)	Mean differences (CI)	ES (CI)
Absolute muscle volumes				
Quadriceps (cm^3^)	1461 ± 238	2309 ± 200**	849 (622 : 1075)	3.88 (2.27 : 5.50)
Hamstrings (cm^3^)	688 ± 98	1125 ± 151**	437 (304 : 571)	3.39 (1.90 : 4.87)
Adductors (cm^3^)	803 ± 140	1268 ± 191**	464 (288 : 640)	2.75 (1.42 : 4.08)
Normalised muscle volumes				
Quadriceps (cm^3^·kg^-1^·m^-1^)	15.74 ± 2.20	17.44 ± 1.78	1.70 (0.36 : -3.75)	0.86 (-0.14 : 1.85)
Hamstrings (cm^3^·kg^-1^·m^-1^)	7.44 ± 1.01	8.45 ± 0.56*	1.01 (0.18 : 1.83)	1.26 (0.22 : 2.30)
Adductors (cm^3^·kg^-1^·m^-1^)	8.66 ± 1.20	9.51 ± 0.85	0.85 (-0.21 : 1.91)	0.83 (-0.17 : 1,82)
Sprint performance				
40m (s)	6.12 ± 0.15	5.25 ± 0.11**	-0.88 (-0.75 : -1.02)	-6.68 (-9.12 : -4.24)
80m (s)	11.07 ± 0.39	9.43 ± 0.26**	-1.67 (-1.34 : -1.99)	-5.01 (-6.95 : -3.08)
Sprint mechanical properties				
F_0_ (N.kg^-1^)	7.42 ± 0.38	8.82 ± 0.90**	1.41 (0.67 : 2.14)	1.98 (0.82 : 3.14)
V_0_ (m.s^-1^)	7.91 ± 0.26	9.76 ± 0.27**	1.85 (1.57 : 2.13)	6.97 (4.44 : 9.50)
P_max_ (W.kg^-1^)	14.67 ± 1.06	21.52 ± 2.29**	6.85 (4.96 : 8.74)	3.76 (2.18 : 5.34)

F_0_, theoretical maximal horizontal force. V_0_, theoretical maximal horizontal velocity. P_max_, theoretical maximal horizontal power. 10m, time achieved in 10m sprint, 40m time achieved in 40m sprint. Data are means ± SD, mean differences ± 95% confidence interval (CI) and effect size (ES) ± 95% CI.

* Significant difference between groups at *P* < 0.05.

** Significant difference between groups at *P* < 0.01.

### Sprint performance

Regarding sprint performance, males were much faster than females over both distances, with extremely large ESs: 40 m (14%, *P* < 0.001), 80 m (15%, *P* < 0.001) (Table 3).

### Sprint mechanical properties

**Fig 3:**
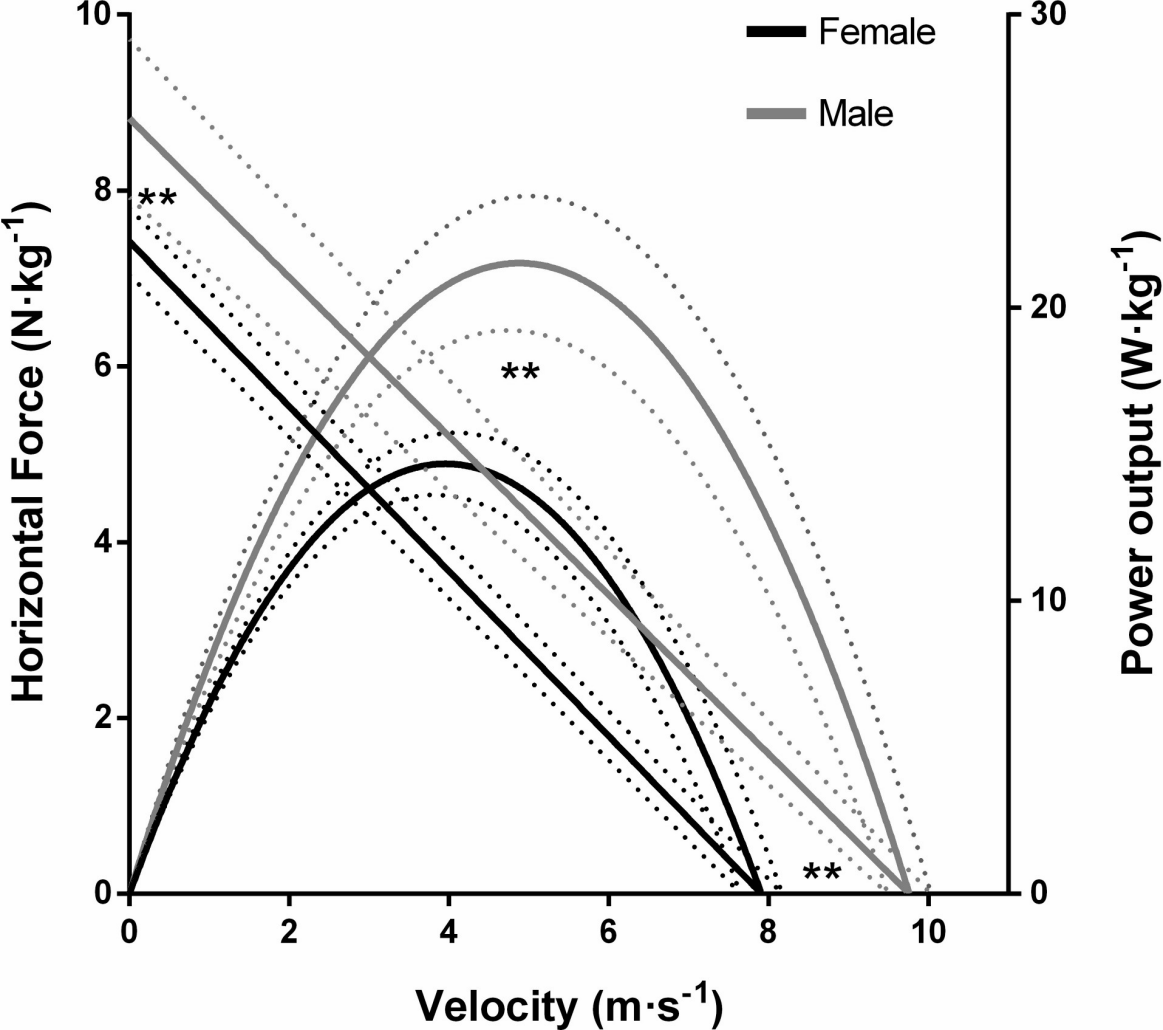
Sprint power–force–velocity profiles of female and male sprinters. Bold lines represent average data. Dotted lines represent standard deviations. ** Significant difference between groups at *P* < 0.01 in F_0_, V_0_ and P_max_.

Males also showed increased sprint mechanical properties, with a larger F_0_ (19%, *P* < 0.01), much larger P_max_ (46%, *P* < 0.001), and very much larger V_0_ (23%, *P* < 0.001) (Fig 3).

### Correlation analysis

**Fig 4:**
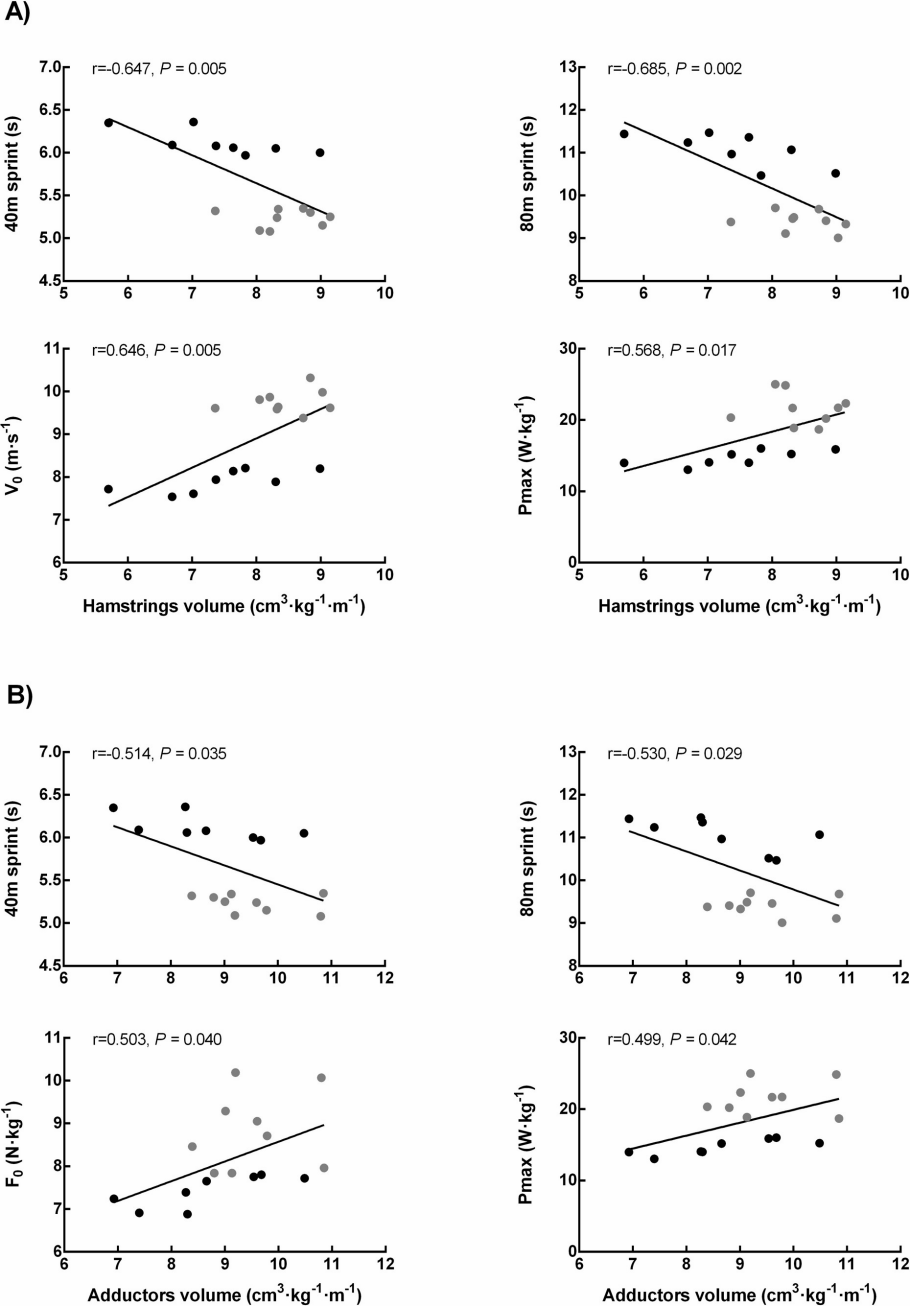
**A) Association between hamstring muscle volume and both sprint performance and mechanical properties. B) Association between adductor muscle volume and both sprint performance and mechanical properties.** R: Pearson’s correlation coefficient. V_0_: theoretical maximal velocity. P_max_: maximal power output. F_0_: theoretical maximal anteroposterior force. Black circles represent female sprinters. Grey circles represent male sprinters.

Normalized hamstring MV correlated strongly with 40 m sprint time (r = -0.647, *P* < 0.01), 80 m sprint time (r = -0.685, *P* < 0.01) and V_0_ (r = 0.646, *P* < 0.01); and moderately with P_max_ (r = 0.568, *P* = 0.017) (Fig 4). No correlation was found between normalized hamstring MV and F_0_ (r = 0.455, *P* = 0.066). The sex-stratified analysis revealed that normalized hamstring MV was correlated to sprint performance only in female sprinters: 40 m sprint time (r = -0.747, *P* = 0.033), 80 m sprint time (r = -0.707, *P* = 0.050), V_0_ (r = 0.711, *P* = 0.048) and P_max_ (r = 0.724, *P* = 0.042). No correlations were found between normalized hamstring MV and sprint performance in males (r = -0.236, *P* = 0.542); however, absolute hamstrings MV was correlated with 80 m sprint time (r = -0.771, *P* = 0.015).

Normalized adductor MV correlated moderately with 40 m sprint time (r = -0.514, *P* = 0.035), 80 m sprint time (r = -0.530, *P* = 0.029), F_0_ (r = 0.503, *P* = 0.040) and P_max_ (r = 0.499, *P* = 0.042) (Fig 4). No correlation was found between normalized adductor MV and V_0_ (r = 0.440, *P* = 0.077). Regarding the sex-stratified analysis, in females, normalized adductor MV was correlated with 40 m sprint time (r = -0.650, *P* = 0.079), 80 m sprint time (r = -0.671, *P* = 0.066), F_0_ (r = 0.761, *P* = 0.028) and P_max_ (r = 0.812, *P* = 0.014). Contrastingly, in males, normalized adductor MV was only correlated with 40 m sprint time (r = -0.733, *P* = 0.039).

Correlation analysis was performed with pooled data (n = 17).

No correlations were found between normalized quadriceps MV and any sprint performance or mechanical property variables (r = -0.401–0.391, *P* = 0.286).

## Discussion

In the present study, we analyzed thigh MV, sprint mechanical properties and performance of male and female sprinters; and also the relationship between thigh MV and sprint performance. The main findings were; 1) males showed larger hamstring MV, while differences in quadriceps and adductors were unclear, 2) males were very much faster than females and exhibited greater sprint mechanical properties, especially maximal velocity, and 3) strong correlations were found between hamstring MV and sprint performance, and moderate correlations between adductor MV and sprint performance, with the pooled data.

Males had very large thigh MVs compared with females (Table 3). MV has been shown to be related to the length of the segment and the mass of the muscle [18,24], and sizes of the segments are directly related to total size and mass of the human body [24]. In this regard, the two groups showed very different anthropometric characteristics (Table 2): males were taller, heavier and leaner, which translates into greater MVs making their comparison unfair. When normalized to body size, these differences were not so clear. While hamstrings were still larger in males, quadriceps and adductors showed non-significant differences (Table 3 and Fig 2); however, a more accurate interpretation would be that differences are uncertain due to the small sample size, which led to such a wide CI, and that a difference between sexes likely exists given the large mean differences and ESs. To our knowledge, very little literature has focused on sex differences in sprinters’ MVs. Handsfield et al. [18] analyzed a group of male and female elite sprinters and found differences in absolute MVs. Nevertheless, when MVs were normalized by height–mass, they found no differences in any lower-limb muscle, concluding that although females were shorter than their counterparts, MVs were proportional to body mass and height. Our results are in agreement with those of Handsfield and coworkers for quadriceps and adductors, but not for hamstrings. Notwithstanding, we should interpret the results of Handsfield et al. [18] carefully, since they based their conclusions only on the significance level, they did not report ESs for each sex. Moreover, they had a small sample size (eight females and seven males) which can directly affect the level of significance.

As expected, males were faster than females, presenting extremely large differences (Table 3), and they also exhibited greater sprint mechanical properties with large to extremely large differences (Table 3 and Fig 3). In sprint events, the differences in performance between males and females are around 10%, when performance level is matched [12], however, in our study, we found differences of 15%. It must be noted that these 10% differences reported by Cheuvront et al. [12] are based on world records and they change repeatedly, and, more importantly, they are not representative of the different performance levels worldwide. For example, the women 100 m world record (10.49 s) established by Florence Griffith-Joyner, dates from 1988. After thirty years the record still remains the same, and since then, the fastest 100 m race run by a woman has been 10.64 s, this is almost 0.2 s slower, and this in sprint terms represents two different performance levels. This fact supports the idea that the actual difference between males and females is probably not 10%, but rather a bit higher and makes the sex comparison based on world records hard to extrapolate to different levels. Anyhow, aside from differences in performance that are intrinsic to gender, the males in this study seem to belong to a slightly superior sprint level. In addition, Slawinski et al. [17] compared the sprint mechanical properties of women and men during 100 m finals of international events over the past 30 years, in order to understand the origins of differences in sprint performance. They found significantly higher F_0_ (10.9%), V_0_ (9.5%) and P_max_ (19%) in males, concluding that the differences in sprint performance between the sexes are due to a lower capability to produce force at high speeds in women, which in turn results in shorter acceleration phases, lower maximal speeds and longer deceleration phases [17]. As shown in Table 3 and Fig 3, males in our study showed greater differences in sprint mechanical properties. Along the same lines as the sprint performance, Slawinski and coworkers based their results on world records, while the athletes in our study belong to a lower performance level. Moreover, it is worth stating that the results of Slawinski et al. [17] were taken from the times supplied by the IAAF, over 30 years, and with different methods. In contrast, data in our study were collected from sprints; thus, comparisons will at least be difficult.

Several morphological characteristics, such as a taller frame, greater muscle mass, larger stride length and higher center of gravity contribute to the male performance advantage in sprinting [11,17]. It has been suggested that faster top speeds are achieved with greater ground reaction forces [15,16]; the larger absolute muscle mass that males possess represents an advantage when it comes to generating these forces [11,15]. Because sprint speed is also affected by step length and step frequency, the longer limbs and the increased muscularity of males contribute to a longer stride which also leads to a faster speed [11,17]. Additionally, marked structural differences in muscle and tendon stiffness have been reported [14,17], being greater in males than in females. It is believed that lower-limb stiffness could influence sprint performance due to the major role played by stiffness in the stretch-shortening cycle [16,17,28]. In addition to the anthropometric and structural differences, studies of muscle characteristics have demonstrated that females have lower muscle glycolytic enzyme activities, as well as smaller cross-sectional type II fibers, than men; and this could also influence performance [14,29].

Strong correlations were found between hamstring MV, sprint performance and V_0_ (Fig 4). Hamstrings have been reported to be key in producing propulsive forces in sprinting [30,31]. In recent literature it has been reported that hamstring MV is a differential factor between sprinters and non-sprinters [10,18]. Moreover, our results add to previous literature correlating hamstring MV with sprint performance [9], which confirms the crucial role of this muscle group in sprinting. Adductors have also been reported to be important during sprinting [6,7,30]. Adductor magnus acts, in fact, as an important hip extensor, and the rest of adductors (pectineus, gracilis, adductor brevis and longus) as hip flexors [30,32]. We found moderate correlations between adductor MV and sprint performance and F_0_ (Fig 4). Interestingly, adductor MV showed no correlation with V_0_. From these results, two different assumptions can be made. First, hamstring muscularity might be more important than adductor muscularity in relation to sprint performance. As seen in Fig 4, hamstring MV showed stronger associations with sprint performance. Second, adductors may play a more important role in the acceleration phase instead of maximum velocity phase, since F_0_ represents the ability to produce horizontal force at very low velocities; in contrast, V_0_ represents the ability to produce force at high velocities [2,17]. Thus, it seems that V_0_, or a “velocity-oriented” profile, is the main mechanical determinant of performance in sprinters [3,17]. It must be noted that the correlations discussed above were performed with the pooled data (males and females together). The sex-stratified analysis revealed that females showed the same associations as the results for the pooled data show (see Results section), supporting our assumption that hamstring muscularity might be more closely related with maximum speed phases and adductor muscularity more involved with the acceleration phase. However, the analysis performed with males showed poor associations between hamstring and adductor muscularity and sprint performance. This could easily be a reflection of the small sample size, which affects the level of association, and which is the greatest limitation of the present study. Either way, our analysis with the pooled data clearly reflects that hamstring and adductor muscularity are related to sprint performance and they could be a differential factor when analyzing sub-groups of sprinters of different performance levels.

## Supporting information

S1 Table Individual data from each volunteer. F_0_, theoretical maximal horizontal force. V_0_, theoretical maximal horizontal velocity. P_max_, theoretical maximal horizontal power. 10m, time achieved in 10m sprint, 40m time achieved in 40m sprint.
(DOCX)
