# Supplementary material for: Sex differences in thigh muscle volumes, sprint performance and mechanical properties in national-level sprinters
Source: PLoS One. 2019 Nov 5;14(11):e0224862. doi: 10.1371/journal.pone.0224862 (PMC6830821; doi:10.1371/journal.pone.0224862)
Supplement: S1 Table — F0, theoretical maximal horizontal force. V0, theoretical maximal horizontal velocity. Pmax, theoretical maximal horizontal power. 10m, time achieved in 10m sprint, 40m time achieved in 40m sprint. (DOCX) [file pone.0224862.s001.docx]

**Individual data from each volunteer**

|  | **Height (m)** | **Body mass (kg)** | **Muscle Volume QUAD (cm^3^)** | **Muscle Volume HAMS (cm3)** | **Muscle Volume ADD (cm3)** | **40m sprint time (s)** | **80m sprint time (s)** | **F0 (N·kg^-1^)** | **V0 (m·s^-1^)** | **Pmax (W·kg^-1^)** |
| --- | --- | --- | --- | --- | --- | --- | --- | --- | --- | --- |
| **Female01** | 1,54 | 52,3 | 1437 | 615 | 668 | 6,06 | 11,36 | 6,88 | 8,14 | 14,00 |
| **Female02** | 1,64 | 52,5 | 1352 | 604 | 712 | 6,36 | 11,47 | 7,39 | 7,61 | 14,05 |
| **Female03** | 1,62 | 52,3 | 1347 | 763 | 810 | 6,00 | 10,52 | 7,75 | 8,20 | 15,89 |
| **Female04** | 1,55 | 50,2 | 1073 | 573 | 674 | 6,08 | 10,97 | 7,65 | 7,94 | 15,19 |
| **Female05** | 1,73 | 68,7 | 1590 | 677 | 822 | 6,35 | 11,44 | 7,24 | 7,72 | 13,98 |
| **Female06** | 1,71 | 57,9 | 1347 | 664 | 734 | 6,09 | 11,24 | 6,91 | 7,54 | 13,03 |
| **Female07** | 1,59 | 56 | 1750 | 739 | 934 | 6,05 | 11,07 | 7,72 | 7,89 | 15,23 |
| **Female08** | 1,67 | 66,4 | 1789 | 868 | 1073 | 5,97 | 10,47 | 7,80 | 8,21 | 16,01 |
| **Male01** | 1,70 | 66 | 2458 | 976 | 1214 | 5,35 | 9,68 | 18,47 | 9,38 | 18,68 |
| **Male02** | 1,76 | 67 | 2022 | 1079 | 1063 | 5,25 | 9,33 | 17,38 | 9,62 | 22,34 |
| **Male03** | 1,89 | 88,4 | 2648 | 1509 | 1635 | 5,15 | 9,01 | 16,51 | 9,98 | 21,72 |
| **Male04** | 1,87 | 79,9 | 2528 | 1100 | 1254 | 5,32 | 9,38 | 17,46 | 9,61 | 20,33 |
| **Male05** | 1,83 | 70,5 | 2198 | 1073 | 1175 | 5,34 | 9,49 | 17,78 | 9,64 | 18,88 |
| **Male06** | 1,83 | 74,3 | 2260 | 1091 | 1248 | 5,09 | 9,71 | 18,50 | 9,81 | 25,00 |
| **Male07** | 1,76 | 69 | 2130 | 1074 | 1069 | 5,30 | 9,41 | 16,89 | 10,32 | 20,22 |
| **Male08** | 1,76 | 73 | 2239 | 1069 | 1233 | 5,24 | 9,46 | 17,31 | 9,59 | 21,69 |
| **Male09** | 1,85 | 76 | 2300 | 1155 | 1519 | 5,08 | 9,11 | 17,59 | 9,87 | 24,85 |
